# Understanding complex causes of suicidal behaviour among graduates in Bangladesh

**DOI:** 10.1186/s12889-024-17989-x

**Published:** 2024-02-22

**Authors:** Jarin Tasnim Tasfi, Shafi Md Mostofa

**Affiliations:** Department of World Religions and Culture, University of Dhaka, Arts Building, 1000 Dhaka, Bangladesh

**Keywords:** Suicide, Family structure, Unemployment, Education, Graduate

## Abstract

This study utilizes both fieldwork and desk-based discourse analysis of newspaper reports to investigate the concerning number of suicides among graduates in Bangladesh. According to some reports, a majority of suicide cases involve young adults who are either currently studying at university or have recently completed their degree (between the ages of 20 and 32). This research contends that patriarchal social expectations in Bangladesh place significant pressure on young adults to secure well-paying jobs to support their families and uphold their family’s status, which can have a negative impact on their mental health. Furthermore, this article identifies additional risk factors that contribute to the high suicide rates among graduates in Bangladesh. These factors include unemployment, poverty, relationship problems, drug addiction, political marginalization, and the stigma of shame, all of which can cause low self-esteem and suicidal thoughts. Moreover, the research suggests that families in Bangladesh have not been providing adequate support to their young members when facing challenges in life. On the contrary, families have added to the pressure on young adults, which can be attributed to joiner’s theory of the effect of industrialization on family norms and values.

## Introduction

Suicide is a worldwide issue, with 703,000 people taking their own lives annually, according to the World Health Organization [1]. This problem is particularly severe in low-income and middle-income countries like Bangladesh, where 77% of all suicides occur [1]. It is worrying to note that nearly 20% of suicides go unreported [2]. Asians account for up to 60% of global suicide cases, with the suicide rate being higher in this region than any other [1, 3]. The impact of each suicide death is substantial, affecting approximately 5–6 people, leading to an annual impact of more than 60 million people worldwide. Despite this, suicide receives less attention in Asia compared to the West [4]). Epidemiologic patterns, risk factors, and protective factors of suicide in Asia differ significantly from those in Western countries, and hence are the primary sources of suicide literature [5]. Therefore, suicide is still a neglected global public health issue, and more attention and resources should be devoted to addressing this problem in Bangladesh and other low-income and middle-income countries. According to the Bangladesh Bureau of Statistics, there were 11,000 reported cases of suicide in 2021, indicating a high suicide rate in Bangladesh [6]. The number of suicide attempts among university graduates in the country has been rising at an alarming pace. A report published by the Aachol foundation on The Dhaka Tribune reveals that at least 101 university students committed suicide in 2021, with 64.36% being male students. Furthermore, 49% of the suicide deaths occurred in the 20–35 age group. In 2022, the foundation recorded 532 suicide cases, with a majority of the cases being students [7]. As a result, this study aims to uncover the reasons behind the increasing number of graduate suicides in Bangladesh.

That said, Durkheim’s work is considered the most significant sociological contribution to understanding the problem of suicide [8]. He believed that explanations based solely on individual psychology were inadequate, and suicide should be viewed as a social phenomenon influenced by sociological factors. According to his theory, suicide rates differ based on sociological group characteristics, such as individuals living alone or in big cities and those with weak social ties. Joiner also emphasizes social issues, asserting that a lack of social connection and experiences of abuse, pain, and previous suicidal thoughts increase the likelihood of suicide [9]. While Durkheim focused on sociological factors, Freud highlighted the psychological aspect of suicide, suggesting that repressed desires to kill an ambivalently regarded lost love could contribute to suicidal thoughts [8, 10]. Many studies have explored the various factors contributing to suicide worldwide, identifying biological, psychological, and social factors as potential causes of suicidal ideation and behaviors.

Sinyor et al. identify the global financial crisis as a significant factor, along with a history of self-harm and parental loss or separation [11]. The cause of suicide is often attributed to a combination of factors, including feeling like a burden on loved ones, experiencing a sense of isolation, and, disturbingly, having learned the ability to self-harm [12]. According to a global study conducted, depression and substance use are major contributing factors to suicide worldwide [13]. Adult suicide has also been linked to loneliness and rejection by a lover [14]. Much global research has linked marital status to suicide. Married people have a decreased suicide risk than singles, divorcees, and widows [15]. However, Mashreky disagree with the above findings and show that most suicide victims (82%) are married [16]. Some psychological issues such as psychiatric illnesses, substance/alcohol abuse, a prior history of suicide attempts, and an acute life event are identified as risk factors. Physical disease is considered one of the most legitimate causes of suicide among young individuals [17].

The causes of suicide in Asia differ from those in other parts of the world. The cultural, social, and economic contexts of Asian countries may contribute to unique risk factors for suicide. Even though psychological issues affect suicide tendency,suicides in Asia are more likely to be caused by financial problems [18–21]. Other severe life stresses, such as loss of employment, gambling, and work-related stress, are common triggers for Asian men to commit suicide. On the other hand, family conflicts are the most significant cause for Asian women [5, 19]. It is a fact that being married is not always a protective factor against female suicide in Asian families [5]. In his study, Vijayakumar et al. show that in India, dowry disputes are thought to be the cause of 98.7% of female suicides [22].

Some Asian nations consider suicide to be acceptable. Hara-kiri (belly cutting), an honourable and altruistic suicide, is practised in Japan [23]). Suttee was a socially acceptable form of widow self-immolation in India [2]. A study conducted in India reveals that 25% of the participants had severe mood disorders [24]. In Bangladesh, there has been a lack of attention to suicide risk factors in the research literature. Despite the high rates of suicide in the country, there has been limited investigation into the underlying factors contributing to suicidal ideation and behaviour. In their study,Arafat et al. show that the average age of attempting suicide is 26.30 (± 12.36) [25]. According to Bangladesh Mental Health Association, indicators include recurring hopelessness, helplessness, desperation-sleeplessness, social withdrawal, loss of food, lack of interest in customary activities, and a sharp and unexpected shift to a cheerful attitude toward death. Another study suggests that economic and career issues, health issues, and failure in life are some factors that lead to suicide ideation [26].

Interpersonal problems, marital issues, domestic or intimate partner abuse, complex gender role expectations, and culture of extended family structure, among other variables, are viewed as contributing to suicidal behaviour in South Asian culture, including Bangladesh [27]. Shah et al. add that problems in the workplace, financial constraints, extramarital affairs, domestic violence, and physical illness also increase the risk of suicide [28].

When it comes to youth’s suicidal behaviour, it is suggest that most of the Public university students who committed suicide had despair, hopelessness, perfectionism, family problems, relationship breakups, lack of social support, financial difficulties, and academic stress [29]. Bhuiyan et al.add that relationship complexity, academic failure, mental health problems, and sexual complexities were the prominent suicide causalities among youths [30]. Post-traumatic stress disorder (PTSD) symptoms, depression, drugs, physical illness, criminal victimization and domestic violence are also found to be catalysts for suicide ideation in Bangladesh [31].

The literature review section above clearly indicates that suicide is a multifaceted phenomenon influenced by various interconnected factors, as suggested by Ajdacic-Gross’s work [32]. The study acknowledges that suicide can be studied from social, psychological, and biological perspectives. This article specifically delves into the sociological aspect of suicide, drawing on Durkheim’s framework [8]. Consequently, social science methodologies have been utilized to unearth the intricate causes of suicidal behavior among graduate students. Furthermore, the research underscores the lack of investigation into why graduates in Bangladesh are experiencing increased rates of suicide. Hence, this article aims to identify multifaceted factors contributing to suicidal tendencies among university students aged 20–35.

## Methodology

This study adopts an exploratory approach, employing in-depth expert interviews to comprehensively investigate suicidal behaviour. A purposive selection process led to the inclusion of 20 experts renowned for their expertise in suicide prevention, experience in working with students, and substantial research background in suicide and mental health. These experts were chosen purposefully for their diverse skills, aligning with the complexity of the study. The selection of 20 experts aligns with the recommended ideal number [33], and data saturation was reached at this count. Face to face interviews were conducted and they were contacted by email, telephone, and direct contact due to researchers’ easy access to experts. Within this cohort, seven experts hold positions as university teachers actively involved in overseeing graduate well-being as mentors or advisors. Additionally, five possess specialized research knowledge in suicide prevention, six are mental health professionals, and two work in humanitarian organizations. Employing a semi-structured questioning format during interviews allowed for comprehensive insights into expert opinions, a crucial tool in understanding social facts within social science research [34].

Interviews were meticulously transcribed, and recurring themes, concepts, and patterns were methodically identified in the responses. Thematic analysis was employed to systematically identify, analyze, and present these patterns, ensuring their reliability through constant comparison and refinement. This process facilitated the recognition of commonalities, differences, and unique perspectives across various interviews. To triangulate primary data, secondary literature and newspaper articles were utilized. Specifically focusing on real case studies within Bangladesh, keywords such as “graduate,” “suicide,” “depression,” “mental health,” “unemployment,” and “Bangladesh” were employed in databases like PubMed, ProQuest, and Google Scholar to extract relavant findings. Suicide notes published in newspapers were also analyzed to understand the multifaceted factors contributing to suicide. While this research aimed to include insights from victims’ family members, financial constraints limited our ability to engage a larger number of respondents. Last but not the least, this research project was approved by the higher studies board of the Department of World Religions and Culture, University of Dhaka, but waived the need for consent to participate and informed consent.

### Theorizing suicidal ideation and behaviour

Durkheim pioneered theories for understanding the underlying causes of suicide, emphasizing that social facts play a significant role in shaping suicidal behaviour. To him, social integration measures individuals’ degree of immersion and shared beliefs with their peers. Social regulation reflects the restraint imposed by societal norms and regulations on individual behaviour [35]. Durkheim explained that egoistic suicide occurs when an individual is socially disconnected, resulting in weak adherence to societal norms and rules, proposing that social groups, such as families, churches, or peers, serve as a strong defense against suicide. Insufficient bonding or connection with society, family, or friends can reduce self-esteem and elevate the likelihood of suicide. Anomic suicide is caused by the absence of regulation in society, which can lead to feelings of disconnection and hopelessness during times of stress and frustration, while fatalistic suicide arises from excessive regulation and high societal expectations, leaving individuals with no agency or control over their lives [8].

Durkheim contends that modern industrialized societies have a more significant potential for anomie. His book *Suicide* suggests that the breakdown or disruption of the family and economic crises occur more frequently in industrialized societies. As industrialization engenders heightened desires and appetites in modern societies, industrialization has become the ultimate objective for individuals and societies without constraining authority. Consequently, he pointed out that an industrialized society has heightened the probability of family breakdown, intermittent economic turmoil, insatiable needs and wants, and social inequality. Industrialization promotes individualism, leading to a decline in traditional family values. This results in the family becoming less cohesive and family members becoming more isolated and independent from one another.

The lower family cohesion caused by industrialization is deeply connected with suicide [36]. The absence of strong familial and community ties can serve as a factor for suicide, erasing a valuable support system during times of crisis. People who are well-integrated with their families and communities benefit from this support system, which helps them cope with adverse life events. research show that low-income family bonding, lack of parental supervision, and limited activities with parents were linked to suicidal ideation [37]. Yadegarfard et al. [38] agree with Bearman and Moody. Family issues, such as marital conflict, poor parenting styles, and physical and sexual abuse, are potential risk factors for suicide [39].

Family or social support appear to be an effective way to prevent suicide. Kleiman and Liu show that social support can lower the chances of suicide attempts [40]. The supportive environment provided by families can help individuals cope with adverse life events, decrease feelings of isolation and loneliness, and enhance their sense of belongingness and thereby preventing individuals from suicide.

Family support could have been fundamental to reducing the possibility of suicide. Instead, the industrial societies and family system have placed high expectations and demands on graduates resulting in personal life crises. Financial stability can be a significant pressure source for graduates, particularly those from low-income families. Poverty can create challenges for them, such as access to education, employment, and other social sectors, affecting mental health and well-being. Relative deprivation and poverty as forms of strain that can cause psychological problems, including substance misuse, alcoholism, and other deviant behaviours that may lead to suicide [41]. When an individual living in extreme poverty observes others from similar backgrounds enjoying significantly better lives, they experience a form of deprivation strain Rehkopf and Buka find a link between poverty and suicide [42]. The authors found a consistent association between socio-economic factors, including poverty, unemployment, income inequality, and suicide rates. The study indicated that areas with higher poverty rates had increased rates of suicide and hopelessness. Above findings that poverty might exacerbate feelings of hopelessness and despair, thus increasing the risk of suicide [43].

Instead of supporting graduates, the industrial society pressures them to find suitable jobs. In this regard, research by Catalano et al. are very relevant. They demonstrate that unemployment significantly impacts mental health, which in turn increases the risk of suicide. Unfavourable financial and career situations can lead to psychiatric and behavioural disorders, including suicide [44]. Unemployment, financial stress, loss of income, and job insecurity can contribute to feelings of hopelessness, despair, and low self-esteem, which can further increase the risk of suicidal behaviour [44]. Low self-esteem raises the risk of having suicidal thoughts and attempting suicide [45]. Low self-esteem or hopelessness also creates psychological pain or depression, which is a crucial factor in the emergence of suicidal ideation and behaviour [46, 47]. Mental suffering increases the likelihood of suicidal ideation [48].

Drawing from the concept of social facts and their impact on mental health, this article contends that the patriarchal society in Bangladesh places immense pressure on graduates to secure a job after graduation, resulting in anxiety and, in some cases, suicide. Furthermore, other factors such as relationship breakdowns, bereavement, poverty, mental illness, and political failures contribute to depression among young people.

### Result

Collected data has been analysed thematically. They are presented below:

### Lack of family support

Increasing evidence suggests that suicide risk is affected by social changes. Industrialization and urbanization have led to families becoming more nuclear and parents becoming more focused on work. In Bangladesh, this has resulted in a lack of communication and support between parents and their children, leaving younger generations feeling lonely and unsupported during times of crisis. Interview no. 4 highlights the difference between the past extended families and the present nuclear families, where parents are too busy to provide practical guidance and support to their children [13 July 2022]. Troll also suggests that the lack of communication in nuclear families exacerbates feelings of loneliness [49]. Interview no. 7 expresses fear about the deteriorating relationships between the current generation and their family members [4 August 2022]. Interview no. 4 explains that nowadays, parents are only conscious about success and career. She adds that “children face constant pressure from parents to achieve high levels of success in their academic and career pursuits” [13 July 2022]. Interview no. 2 notes that the ratio of male suicide is higher in Bangladesh. He points out the social structure of our community.

In a patriarchal society, the masculine gender role ideology often stipulates that men must conform to traditional masculine norms of strength and dominance. It creates psychological distress, including feelings of inadequacy, shame, and anxiety, when a boy fails to meet these societal expectations. The typical expectations of becoming the leader of the family harden the situation. Moreover, the socio-cultural situation of Bangladesh expects men to be emotionally strong, which often creates a mindset that prevents them from taking mental health support [14 July 2022].

Instead of putting pressure on graduates, family members can provide crucial support during periods of stress to reduce suicide [50]. Harris and Molock found that stronger family cohesion and support are associated with a lower likelihood of suicidal thoughts [51]. Interview no. 11 also highlights that family problems could lead to hopelessness, anxiety and frustration by pointing out a story where a graduate died by suicide as he could not cope with his family crises [September 1 2022].

### Unemployment

The process of industrialization has brought about significant changes in the economy, such as the expansion of manufacturing industries, improved transportation and communication systems, and increased access to goods and services, leading to overall economic growth and development. However, it is crucial to note that economic factors have a significant impact on suicidal ideation, and industrialization alone cannot solve all economic crises. Analyzing the economic situation of Bangladesh can help identify the reasons behind the alarming suicide rates. According to official World Bank figures, Bangladesh’s GDP in 2021 was approximately 416.26 billion U.S. dollars, accounting for 0.3% of the world’s economy [52].

Additionally, Bangladesh ranked 116 out of 167 countries in living conditions, according to the Legatum Prosperity Index 2021 [53]. The average earning is 61.1 taka (2016) or US$1.90 (2011 PPP) per day per capita, as per the international poverty line scale. The population size of Bangladesh is currently 165,158,616, with a population density of 1119 per km2, making it the 8th most populated country globally [54]. As per the Population and Housing Census 2022 report, the total percentage of youth in Bangladesh is approximately 17.79% of the total population (age limit 20–29). The world bank report also indicates that the unemployment rate is 5.20% (2021), which is a consequence of overpopulation( World Bank, 2021)52. However, according to the Bangladesh Bureau of Statistics (BBS), the unemployment rate in Bangladesh was 10.6% in 2021, more than twice the national average unemployment rate of 5.2% [55].

Respondent no. 6 expresses concerns over the passion for BCS (Bangladesh Civil Service) in Bangladesh. “There are few limited recruitments. Most of the candidates do not have a backup plan. Soon they become anxious and frustrated” [1 August 2022]. Respondent no. 7 states that the government job requirement is not only a long process but also has an age limit, which puts pressure on graduates [4 September 2022]. A 2022 report reveals that 2,76,760 people applied for 1,710 vacancies in the 44th BCS held on May 27, 2022 [56]. The examination is conducted in three phases. Another report in the Daily star shows that the 38th BCS required at least two to three years to finish the recruiting process, which includes a thorough police check, health examination, and gazette publication of the outcome [57]. This is a waste of young people’s potential that frustrates many individuals. Respondent no. 4 shares a story of a victim who died by suicide due to failure to secure a good job.

I knew a brilliant student who tragically ended his life. As a graduate in a high-demand field, he faced immense pressure to meet the expectations placed upon him. He aspired to secure a prestigious government job, but his hopes were shattered when the results of the competitive job exam were announced. The disappointment was so overwhelming that he fell into a deep depression and ultimately took his own life. According to his friends, the failure to meet these job requirements was the catalyst for his intense frustration and subsequent despair [13 July 2022].

While government job scarcity is alarming, we cannot undermine the scarcity of relevant jobs in Bangladesh. Interviewee no 6 states that the University of Dhaka has 83 departments, and most do not offer any applicable job sector for their graduates. Most non -governmental jobs demand specific college degrees such as engineering, MBA, or development studies [August 1 2022]. Complying with these findings, Roy et al.that most people need more technical knowledge and skills to keep up with the latest technological developments[58]. On the other hand, people with general education are rendered incapable of accomplishing any work [59]. Bangladesh’s high unemployment rate hinders economic growth. Bangladesh’s economy can grow with a young, educated population [60]. A deep connection exists between increased unemployment and suicide rates [44]. Unemployed persons tend to participate in unhealthy behaviours and lifestyles, which raise depression risk [61, 62]. Montgomery et al. show that unemployment is similarly associated with anxiety risk [63].

### Poverty

Financial instability and poverty can significantly increase suicide risk. Income and wealth equality (affluence or poverty) safeguard against suicide. The association between suicide and socio-economic factors, such as poverty, financial crises, indebtedness, and unemployment, is consistently documented in the scholarly literature [64, 65, 66]. Interview no. 1 explains two types of poverty: actual poverty and relative deprivation. Relative deprivation can have a significant impact on the youth. Despite having access to necessities such as food, clothing, and shelter, they often feel deprived compared to others who are more privileged and achieve success quickly. Constantly witnessing these disparities can negatively affect their cognitive functions, including perception, attention, memory, decision-making, and psychological well-being [7 July 2022].

Poverty is a significant concern for the people in Bangladesh. Interview no. 9 shares a story where he links poverty with unemployment. A graduate who belonged to a highly underprivileged background opted for death by suicide because he was unable to find a job. He stated that “after several failed attempts to find a job, he felt that he would never be able to lead a financially stable life, his financial background took his depression to a serious level, and he decided to end his life. Only if he could have waited for a little more, he would have been able to find a job to improve the situation” [17 August 2022]. Respondent no. 4 argues that many public university students belong to the lower income close. They feel that the sooner they get a job, the more they can support their family [13 July 2022].

### Peer pressure

Upon completing their formal education, students enter a transitional period where they seek stability in their personal and professional lives before entering the workforce or pursuing higher education. Financial stability is a crucial factor for many, and peer pressure can exacerbate stress levels, anxiety, and depression among young people. All respondents agreed that peer pressure was a significant factor for graduates. Studies have linked peer pressure to increased stress, anxiety, and sleep problems in young adults [67]. Respondent no. 6 also noted that social pressure, such as getting married and starting a family, can lead to depression, particularly when a young person observes their peers settling into their own lives [1 August 2022].

### Uncomfortable relationships

Relationship breakups and hostile relationships can lead to suicidal thoughts and actions. According to Whisman and Baucom, Individuals’ mental health is significantly impacted by their intimate partner’s interactions [68]. In addition, suicidal ideation is significantly influenced by terrible life circumstances mainly caused by a spouse or partner [69]. Interpersonal conflict [70] and separation [71] are two primary triggers for suicide. Separation is stressful or upsetting, which may lead to an aversion to challenges or pain, strengthening the acquired ability to engage in self-harm behaviour [9]. Interviewees have suggested a troubled relationship. Respondent no 7 said, “When young people enter into romantic relationships, they face unique challenges, such as trials and conflicts, that may result in relationship termination. These experiences can profoundly impact their mental well-being, as young people tend to make emotional decisions and struggle to reason during trouble” [September 4 2022].

When a relationship ends, they may feel like their life has reached a standstill and lose motivation to continue [72]. Several cases suggest that youth graduates chose suicide due to frustration over relationship breakups. For instance, in 2022, a report revealed that a graduate committed suicide after breaking up with his girlfriend and leaving behind a suicide note [73]. This situation highlights the impact of relationship breakups on young people’s mental health and their susceptibility to suicidal ideation.

#### Loss of dearest one

The loss of a loved one can cause a significant emotional burden, increasing the risk of suicidal thoughts and behaviours. The psychological impact of bereavement can be overwhelming, causing individuals to experience intense sadness, hopelessness, guilt, and anger. According to respondent no. 10, losing a loved one can lead to anxiety attacks, chronic fatigue, depression, and suicidal thoughts [24 August 2022]. A report by Bdnews24 tells the story of a Dhaka University graduate who fell into severe depression after his mother’s death. Unable to cope with the pain, he took his own life [74]. Studies have also linked the unexpected death of a loved one to depression, anxiety, drug use, and other mental disorders, as well as an increased risk of long-lasting grief reactions [75].

### Depression

Psychiatric pain or depression can have severe consequences on an individual’s mental well-being, leading to suicidal thoughts and behaviours. Respondent no. 11 highlights that the lack of understanding of psychological issues among patients is a significant issue that results in delayed recoveries and increased feelings of hopelessness and loneliness, which can often lead to suicidal behaviour. Research has identified psychiatric pain as a crucial factor in developing suicidal ideation and behaviour.Mee et al. found that patients reported psychological distress to be more unbearable than any physical pain they had experienced. Social support is crucial in preventing and coping with the effects of stressful situations brought on by psychiatric pain or depression[76]. people with social support are better equipped to cope with such situations [77].

### Lack of help and stigma

A study shows that 45% of suicide victims contact a primary care physician within one month of death without disclosing suicidal thoughts [78]. Interview no 6 states the scarcity of psychological help [1 August 2022]. In this context, a report in the Daily star stated that the current treatment gap for adults is 92.3%, and for children, it is 94.5%. According to the same survey data, only 400 clinical psychologists serve a population of 16 billion [79]. Interview No. 3 expresses the concern that people with mental illness are often addressed as mad or psycho. They do not want to take help because they fear people will socially exclude them [11 July 2022]. Interview no. 11 also opines that people already suffering from mental health issues are more vulnerable. Sometimes, they even get bullied for the issue. The respondent shares a story of a girl who committed suicide.

A girl suffering from clinical depression was advised by her doctors to rest full-time to recover. However, as she missed lectures at her university, she was required to provide a cause for her absence. When she explained that she was advised to rest, her teacher belittled her illness, stating that it was not real and that the younger generation took such matters too seriously. This comment profoundly impacted the girl, leading her to conclude that she was not worth living. She attempted to kill herself that night by cutting her veins but was fortunate enough to be taken to the hospital in time [1 September 2022].

### Political marginalization

Political marginalization is relegating a group or class of individuals in a given society to secondary or inferior positions. It is an intentional strategy to exclude people of such a society from political arguments, economic negotiating, and social bargaining [80]. Student politics refers to the political actions of students linked with the organization of the student body and their influence on the higher education institution, higher education systems, and society at large [81]. Student politics can also mean acting as a student branch of an active national political force, forming future leaders for that party, and using student power to help that political group. It works on issues important to students, gets adopted as an agenda, and pressures people in power to make sound political decisions about that agenda.

Students played a crucial role in anti-British activities during British rule, the linguistic movement of 1952, the six-point movement of 1966, the mass uprising of 1969, and the liberation war of 1971 [82]. Bangladeshi students have been at the forefront of the nation’s ongoing campaign for democracy for years. However, at present, human rights organizations and activists have harshly criticized the current autocratic regime. The V-Dem Institute, an independent research institute based in Sweden, recently classified Bangladesh as an “electoral autocracy” in its fifth annual democracy report [83]. Mostofa and Subedi call this regime a ‘competitive authoritarian regime’ [84] and Mostofa argues that radical nationalism has grown in Bangladesh. Interview no. 9 shares his observance of political alienation [85].

I knew a group of Dhaka university students who belonged to the student political group in Bangladesh. They were highly active, influential, and regarded as the privileged class of TSC. On January 23, 2018, they protested before the vice chancellor’s office. On January 15, the ruling party’s student wing assaulted female students at a rally that urged the D.U. to scrap its affiliation with seven Dhaka-based colleges. The previous party again badly attacked them. The attack injured at least 30 students, including 10 in critical condition. They never got justice [26 August 2022].

Moreover, their activism eventually faded away. Sadly, three party members took their own lives, one of whom was the most brilliant student in their department, earning first place at graduation. In addition to his outstanding academic record, he was known for his dedication, courage, and intelligence. This occurrence had a severe psychological impact on their mental and physical health [17 August 2022].

A reputed psychiatrist in Bangladesh said that youth’s mental health could be severely impacted by exposure to political unrest and violence, leading to significant anxiety [Interview no. 8, 9 August 2022]. Some studies support this claim. If the collective movement fails to fulfil its goals, frustration may lead to depression [86, 87]. The frequency of anxiety and PTSD following collective acts are equivalent to those experiencing terrorist attacks and armed conflicts, both in the general population and among victims of violence [88, 89, 90, 91]. In a study, suicide was one of the significant findings among the people who actively participated in protests [92]. They also add that political unrest can increase the likelihood of suicidal behaviour. For instance, a political activist from the ruling party in Bangladesh reportedly died by suicide after being physically harassed and insulted by another influential member of the same group [93].

Another example is an active opposition party activist who chose death by suicide. His family said he was so invested in politics that he never pursued a job. The current political situation took a severe toll on his mental health, and his frustration ultimately led him to take his life [94].

### Drug addiction

Interview 11 said that drug addiction is one of the major problems among youths in Bangladesh. It destroys one’s mental stability. “As we know, the post-graduation period is challenging for a person to adapt to new changes; they are more likely to get exposed to drugs” [1 September 2022]. Interviewee 12 shared a personal story about a successful young person committing suicide. “He became addicted to weeds and alcohol because of a personal life crisis” [14 September 2022].

Drug dependence is a chronic brain disorder. It causes a person to consume drugs despite their harmful effects repeatedly. Unfortunately, young individuals have a far higher rate of usage and abuse of prescription drugs than older age groups. Bangladesh has become a high-risk nation for sustainable development due to the growing number of drug users, mostly young. A report published in the Dhaka tribune shows that the city’s narcotics bureau identified three thousand and five hundred drug dealers in Dhaka in 2021 [95]. Meanwhile, According to DNC’s annual report, people aged 16 to 40 comprise 84.27 per cent of the total drug abusers in Bangladesh, and drug abuse is most severe among those aged 21 to 25 [95].

Interviewee 9 has discussed drug addictions. “Some drugs can cause a psychotic episode. This means they have hallucinations, delusions, confusing and unsettled ideas, and little self-awareness. So they have little idea what they are doing [26 August 2022]. According to a report in Dhaka tribune, a graduate died by suicide by slitting his throat. The Dhaka Metropolitan Police Detective Branch identified a link between a student’s death and LSD [96]. Different studies, confirm that substance use disorders (SUD) might be the most investigated and well-known risk factor for suicidal activity [97]. While interviewee 11 also explains that depressive symptoms, certain personality qualities, and specific circumstances could all be shared triggers for substance misuse and suicidal conduct. Addicted and chronic young drug abusers are generally troubled [1 September 2022].

### Suicide contagion

One thing that has been debated for a long time is the effect of seeing other people’s death by suicide (known as exposure to suicidal behaviour, or ESB) called “suicide contagion” [98]. Two respondents point out this factor as well. According to Interviewee 5, the replicating trend is prevalent among the youth. He explained that when they see someone they know commits suicide, they try to analyze their own situation. They try to justify their suicide ideation by assuming the deceased person was in a better situation yet committed suicide [20 July 2022]. Interviewee 11 further adds that suicide is contagious only among those already suffering from frustration, depression, or personal life crisis [1 September 2022].

## Discussion

The emergence of industrialization has resulted in significant societal changes with lasting impacts on the contemporary world. This shift has led to the rise of cities, a new working class, and significant changes to social and economic structures. According to some interviewees, industrialization has also brought about a cultural shift in family structure in Bangladesh, resulting in increased feelings of loneliness and lack of support among this generation. This, combined with overburdening expectations and inadequate mental support, can lead to hopelessness and frustration, ultimately resulting in feelings of worthlessness and self-harm. These findings are supported by studies, which shows a correlation between suicidal ideation and inadequate family bonding, inadequate parental supervision, and limited engagement in activities with parents due to industrialization [37, 49].

Industrialization ostensibly brings new job opportunities. In reality, a good proportion of youths lack job facilities across the globe, especially in developing countries like Bangladesh. It was reported that a Jahangirnagar University graduate chose death by suicide. He was suffering from depression after not getting a job which might have acted as a triggering factor [99]. Interviewee no. 6 shares a story he found in his study that most people having death by suicide cases suffer from anxiety about joblessness. He also said that the job market has always had limited opportunities, and the situation has only worsened. The constant struggle to secure suitable employment often leads to anxiety and stress, which can result in severe depression. Many times, this leads them to contemplate suicide [August 1, 2022]. Through their empirical study, Catalano et al.establish a significant correlation between unemployment and mental health, ultimately augmenting the likelihood of suicide [44].

Lack of job opportunities has a deep connection with poverty, which creates tremendous pressure on unemployed youths. Interviewee no. 1 highlighted poverty and relative deprivation as reasons for youth suicide. Poverty in life can cause higher stress levels, leading to psychological problems in later life due to financial struggles. The stress often makes them feel useless and take their own lives [7 July 2022]. Brinkmann contends that poverty and indebtedness can lead to suicidal ideation [100]. The Financial Express triangulates this claim that a Dhaka University student suffering from poverty and other financial losses committed suicide in 2018 [101].

When society or family could have been an antidote against anxiety and depression caused by the industrial revolution, instead, family or society per se is putting pressure of exceptions on graduates. Society, family, and culture often expect graduates to achieve success without delay. In this context, success refers to meeting the societal standards and expectations of the graduate’s desired achievements. Almost all respondents acknowledge that peer pressure is a significant risk factor in the life of a graduate. Interviewee no 6 said that the expectation of achieving specific life milestones, such as securing a stable job or getting married, can cause individuals to experience isolation, exclusion, and low self-esteem. Failing to conform to societal norms and expectations can also contribute to developing suicidal thoughts [1 August 2022]. Study demonstrate a strong correlation between peer pressure and elevated stress levels, anxiety, and sleep difficulties in youth [67].

Bangladesh’s patriarchal social structure is also causing anxiety for youths. Interviewee no. 2 points out that the patriarchal society of Bangladesh, where males are expected to gain resources and power to become the head of the family. This leaves little room for choice regarding their career and no tolerance for failure. The social and cultural expectations of being the breadwinner place an additional burden on male graduates, resulting in heightened pressure and anxiety. As a result, male suicide is notably higher in Bangladesh, likely due to the socio-cultural expectations placed on men [14 July 2022]. These findings are supported by Durkheim, who posited the concept of Fatalistic suicide as a phenomenon resulting from the imposition of strict regulation and high societal, cultural, or family expectations. This can make individuals feel that their lives are predetermined, leaving them no personal control [8].

Anxiety or depression is one of the root cause of suicide. It can be caused by social facts and can also be caused by some immediate reasons like a relationship breakup, the sudden loss of the dearest one, and failure in social and political movements. Relationship problems, including breakups and fights, can be highly distressing and heart-wrenching for young people who tend to be more emotional [Interviewee no. 7, 12 August 12,022] The Daily Bangladesh reported that a graduate chose death by suicide and left a suicide note, citing frustration and uncontrollable anger after a breakup with his girlfriend as the reason for his action[68]. According to Kazan et al., individuals who have recently terminated a romantic relationship may encounter despair, which may augment their vulnerability to suicidal ideation and behaviour [102]. The failed love affair is one of the primary reasons behind the suicide of Chinese graduate students [103].

Several studies suggest that the death of a loved one is associated with the development of depression, anxiety, substance abuse, and other mental health issues, as well as an increased risk for persistent grief reactions [75]. A story of a Dhaka University graduate who died by suicide was revealed in the newspaper. That report suggests that the victim fell into a deep state of depression following the death of his mother. He opted for death by suicide after struggling to cope with the changed situation [74]. An expert also suggests a strong association between losing a loved one and suicidal ideation [Interviewee 10, 24 August 2022].

Thus, psychological pain appears to be a critical factor in developing suicidal thoughts and actions [46, 47]. A study conducted on graduate students found depression as a significant risk factor among them [104]. Respondent no. 11 emphasizes that psychiatric pain or depression is a significant factor in leading to suicide. A person already struggling with psychological issues may feel overwhelmed by adjustment difficulties, guilt and shame of not getting the desired job, high expectations, lack of opportunities, and inadequate support and motivation. The inability to effectively cope with and express their emotional pain can lead them to consider suicide a way out [1 September 2022].

Failure in political and social movements may also cause anxiety in youths. According to an expert, it appears in Bangladesh that exposure to political unrest and violence and failure in political movements can cause severe anxiety and take a toll on the mental health of the youth [Interviwee 8, 9 August 2022]. Matthies-Boon also suggests a similar argument. To him, if the collective movement fails to fulfil its goals, frustration may lead to depression [87].Ni et al. add that people actively participating in failed protests sometimes commit suicide. Bangladesh also witnessed similar cases [92]. A political activist committed suicide as he was physically harassed and severely insulted by another influential person in the same group. The disturbing incident had a significant mental impact on him, potentially acting as a catalyst for his tragic choice [93]. In another case, an active leader who belonged to the current opposition party committed suicide. His family stated that he was so involved in politics that he never took any job. The current political situation has badly affected his mental health, so his frustrations led to his taking his life [94].

People often seek immediate solutions to alleviate their pain rather than medical help, and drug addiction can be an example. Interviewee no. 11 correlates drug addiction with the challenges and frustrations experienced by graduates [1 September 2022]. Overdosing on drugs can impair normal brain function and may lead to suicidal behaviour. Miller et al. found that drug addiction is responsible for suicide [105]. Some recent cases in Dhaka also testify this case. The Dhaka Tribune reported that a graduate chose death by slitting his throat. The Dhaka Metropolitan Police Detective Branch identified a connection between the student’s death and LSD [96]. It is to be noted here that drug addiction can also stem from depression or psychological distress or may have existed prior to these conditions. Regardless of the cause of addiction, drug addiction increases the risk of suicidal behaviour.

## Conclusion

The article asserts that several potential risk factors contribute to the vulnerability of Bangladeshi graduates, encompassing unemployment, poverty, societal expectations, relationship challenges, drug addiction, anxiety, depression, loss of loved ones, lack of medical support, and disappointment in social or political movements. It is argued that industrialization has significantly altered Bangladeshi society, leading to increased urbanization, a new working class, and shifts in social and economic structures. These changes have triggered a shift in family dynamics, leaving younger generations feeling disconnected and unsupported. Additionally, a lack of job opportunities and poverty contribute to feelings of anxiety and hopelessness, elevating the risk of suicidal thoughts.

Pressure to conform to societal norms, especially in achieving success within specific timeframes, worsens feelings of isolation, exclusion, and low self-esteem. Failure to meet these expectations may further fuel suicidal thoughts. The familial support system, once a potential buffer against such thoughts, faces challenges due to societal and cultural pressures, generating low self-esteem among graduates. With this low self-esteem, various factors, including relationship breakups, sudden loss of loved ones, and social or political disappointments, further compound psychological distress. Regrettably, individuals often seek immediate, albeit harmful, solutions like drug addiction instead of seeking proper medical assistance. These feelings of anxiety, hopelessness, and frustration culminate in feelings of worthlessness and self-harm. That said, Extensive literature reviews and expert interviews emphasize the immediate necessity for policymakers to prioritize mental health resources, diminish societal stigma related to mental well-being, and incorporate mental wellness into comprehensive public health strategies, particularly focusing on the youth. It’s pivotal to reconsider the family’s role in nurturing children, ensuring ample support without imposing undue pressure. Likewise, there’s a critical need to reevaluate the education system, equipping individuals with essential skills to navigate life’s complexities effectively.

## Data Availability

Data are stored in the Department with principle supervisor for five years. Professor Dr. Mohammad Jahangir Alam, chairman of the Department of World Religions and Culture, can be contacted for accessing materials. He can be reached at mja77@du.ac.bd.

## References

[1] World Health Organization. Suicide. World Health Organization. 2021, June 17. Retrieved March 25, 2023, from https://www.who.int/news-room/fact-sheets/detail/suicide.

[2] Vijayakumar L, Pirkis J, Whiteford H (2005). Suicide in developing countries. Crisis.

[3] Beautrais AL (2006). Suicide in asia. Crisis.

[4] Vijayakumar L, Pirkis J, Huong TT, Yip P, Seneviratne RD, Hendin H (2008). Socio-economic, cultural and religious factors affecting suicide prevention in Asia. Suicide Suicide Prev Asia.

[5] Zhang J, Ma J, Jia C, Sun J, Guo X, Xu A, Li W (2010). Economic growth and suicide rate changes: a case in China from 1982 to 2005. Eur Psychiatry: J Association Eur Psychiatrists.

[6] Ali E, Maksud M, Zubyra SJ, Hossain MS, Debnath PR, Alam A, Chakrabarty PK (2014). Suicide by hanging: a study of 334 cases. Bangladesh Med J.

[7] The Dhaka Tribune. *Study: Bangladesh saw 446 student suicides in 2022*. 2023, January 27; Available from:https://www.dhakatribune.com/bangladesh/2023/01/27/study-bangladesh-saw-446-student-suicides-in-2022.

[8] Durkheim E. Suicide: a study in sociology. Routledge; 2005. Aug 4.

[9] Joiner TA (2007). Total quality management and performance: the role of organization support and co-worker support. Int J Qual Reliab Manage.

[10] Freud S. The theme of the three caskets. InThe Standard Edition of the Complete Psychological Works of Sigmund Freud, Volume XII (1911–1913): The Case of Schreber, Papers on Technique and Other Works 1958 (pp. 289–302).

[11] Sinyor M, Tse R, Pirkis J (2017). Global trends in suicide epidemiology. Curr Opin Psychiatry.

[12] Joiner T. Why people die by suicide. Harvard University Press; 2005.

[13] Ferrari AJ, Norman RE, Freedman G, Baxter AJ, Pirkis JE, Harris MG, Page A, Carnahan E, Degenhardt L, Vos T, Whiteford HA (2014). The burden attributable to mental and substance use disorders as risk factors for suicide: findings from the global burden of Disease Study 2010. PLoS ONE.

[14] Canetto SS, Lester D (2002). Love and achievement motives in women’s and men’s suicide notes. J Psychol.

[15] Smith JC, Mercy JA, Conn JM (1988). Marital status and the risk of suicide. Am J Public Health.

[16] Mashreky SR, Rahman F, Rahman A (2013). Suicide kills more than 10,000 people every year in Bangladesh. Archives Suicide Res.

[17] Dahlen ER, Canetto SS (2002). The role of gender and suicide precipitant in attitudes toward nonfatal suicidal behavior. Death Stud.

[18] Liu IC, Chiu CH (2009). Case-control study of suicide attempts in the elderly. Int Psychogeriatr.

[19] Phillips MR, Yang G, Zhang Y, Wang L, Ji H, Zhou M (2002). Risk factors for suicide in China: a national case-control psychological autopsy study. Lancet.

[20] Amagasa T, Nakayama T, Takahashi Y (2005). Karojisatsu in Japan: characteristics of 22 cases of work-related suicide. J Occup Health.

[21] Wong PW, Chan WS, Conwell Y, Conner KR, Yip PS (2010). A psychological autopsy study of pathological gamblers who died by suicide. J Affect Disord.

[22] Vijayakumar L (2005). Suicide and mental disorders in Asia. Int Rev Psychiatry (Abingdon).

[23] Takai M, Yamamoto K, Iwamitsu Y, Miyaji S, Yamamoto H, Tatematsu S, Yukawa M, Ide A, Kamijo Y, Soma K, Miyaoka H (2010). Exploration of factors related to hara-kiri as a method of suicide and suicidal behavior. Eur Psychiatry.

[24] Vijayakumar L, John S, Pirkis J, Whiteford H (2005). Suicide in developing countries (2) risk factors. Crisis.

[25] Arafat SY, Kar SK, Kabir R (2021). Possible controlling measures of panic buying during COVID-19. Int J Mental Health Addict.

[26] Ara MJ, Uddin MF, Kabir MH (2016). The causes of suicide and impact of society in Bangladesh. Int Res J Social Sci.

[27] Khan MM (2002). Suicide on the Indian subcontinent. Crisis: J Crisis Intervention Suicide Prev.

[28] Shah MM, Ahmed S, Arafat SM. Demography and risk factors of suicide in Bangladesh: a six-month paper content analysis. Psychiatry J. 2017 Oct 10. 10.1155/2017/3047025 PMC5654290 29130035

[29] Urme SA, Islam MS, Begum H, Chowdhury NR. Risk factors of suicide among public university students of Bangladesh: a qualitative exploration. Heliyon. 2022;8(6). 10.1016/j.heliyon.2022.e09659 PMC9194591 35711983

[30] Bhuiyan AI, Sakib N, Pakpour AH, Griffiths MD, Mamun MA (2021). COVID-19-related suicides in Bangladesh due to lockdown and economic factors: case study evidence from media reports. Int J Mental Health Addict.

[31] Rasheduzzaman M, Al-Mamun F, Hosen I, Akter T, Hossain M, Griffiths MD, Mamun MA (2022). Suicidal behaviors among Bangladeshi university students: prevalence and risk factors. PLoS ONE.

[32] Ajdacic-Gross V (2015). Suicide-background, epidemiology, risk factors. Therapeutische Umschau Revue Therapeutique.

[33] Mostofa SM. Islamist militancy in Bangladesh: a pyramid root cause model. Springer Nature; 2021.

[34] Mostofa SM (2021). Understanding Islamist Militancy in Bangladesh. J Asian Afr Stud.

[35] Bearman PS. The social structure of suicide. In Sociological forum 1991 Sep (Vol. 6, pp. 501–24). Kluwer Academic Publishers-Plenum.

[36] Rubenstein JL, Heeren T, Housman D, Rubin C, Stechler G (1989). Suicidal behavior in normal adolescents: risk and protective factors. Am J Orthopsychiatry.

[37] Bearman PS, Moody J (2004). Suicide and friendships among American adolescents. Am J Public Health.

[38] Yadegarfard M, Meinhold-Bergmann ME, Ho R (2014). Family rejection, social isolation, and loneliness as predictors of negative health outcomes (depression, suicidal ideation, and sexual risk behavior) among Thai male-to-female transgender adolescents. J LGBT Youth.

[39] Pathak N, Singh RN, Singh UP (2017). Suicide and suicidality in India: vulnerabilities and resiliency. Suicide.

[40] Kleiman EM, Liu RT (2013). Social support as a protective factor in suicide: findings from two nationally representative samples. J Affect Disord.

[41] Zhang J, Song Z (2006). A preliminary test of the strain theory of suicide. Chin J Behav Med Sci.

[42] Rehkopf DH, Buka SL (2006). The association between suicide and the socio-economic characteristics of geographical areas: a systematic review. Psychol Med.

[43] Maris RW (1969). The sociology of suicide prevention: policy implications of differences between suicidal patients and completed suicides. Soc Probl.

[44] Catalano R, Goldman-Mellor S, Saxton K, Margerison-Zilko C, Subbaraman M, LeWinn K, Anderson E (2011). The health effects of economic decline. Annu Rev Public Health.

[45] Chatard A, Selimbegović L, Konan PN (2009). Self-esteem and suicide rates in 55 nations. Eur J Personality: Published Eur Association Personality Psychol.

[46] Levi-Belz Y, Gvion Y, Horesh N, Fischel T, Treves I, Or E, Stein-Reisner O, Weiser M, David HS, Apter A (2014). Mental pain, communication difficulties, and medically serious suicide attempts: a case-control study. Archives Suicide Res.

[47] Levi-Belz Y, Gavish‐Marom T, Barzilay S, Apter A, Carli V, Hoven C, Sarchiapone M, Wasserman D (2019). Psychosocial factors correlated with undisclosed suicide attempts to significant others: findings from the adolescence SEYLE study. Suicide Life‐Threatening Behav.

[48] Verrocchio MC, Baker AJ, Bernet W (2016). Associations between exposure to alienating behaviors, anxiety, and depression in an Italian sample of adults. J Forensic Sci.

[49] Troll LE (1982). Family life in middle and old age: the generation gap. Annals Am Acad Political Social Sci.

[50] Compton MT, Thompson NJ, Kaslow NJ (2005). Social environment factors associated with suicide attempt among low-income African americans: the protective role of family relationships and social support. Soc Psychiatry Psychiatr Epidemiol.

[51] Harris TL, Molock SD (2000). Cultural orientation, family cohesion, and family support in suicide ideation and depression among African American college students. Suicide Life-Threatening Behav.

[52] World Bank. World development report 2021: Data for better lives. 2021.

[53] Index LP. Legatum Prosperity Index 2021.

[54] Bangladesh Bureau of Statistics. *Population and Housing Census 2022.Bangladesh* ministry of Planning. Retrieved December 5, 2023, fromhttps://bbs.portal.gov.bd/sites/default/files/files/bbs.portal.gov.bd/page/b343a8b4_956b_45ca_872f_4cf9b2f1a6e0/2023-09-27-09-50-a3672cdf61961a45347ab8660a3109b6.pdf

[55] Ahmed, M. *Graduate unemployment: Who’s to blame?* The Daily Star. 2023, January 13. Available from:https://www.thedailystar.net/opinion/views/news/graduate-unemployment-whos-blame-3219261

[56] The Daily Star. *44th BCS preliminary exams: Over 3.5 lakh candidates vying for 1,710 posts*. https://www.thedailystar.net/youth/careers/public-service/news/44th-bcs-preliminary-exams-over-35-lakh-candidates-vying-1710-posts-3033101

[57] Hasan, S. Patience test’ for govt jobseekers.The Daily Star, 2020, June 4; https://www.thedailystar.net/country/news/patience-test-govt-jobseekers-1908845

[58] Roy JS, Das N, Islam TU. Vocational training: Integral policy measure for reducing youth unemployment. The Financial Express, Dec 31, 2021; https://thefinancialexpress.com.bd/views/views/vocational-training-integral-policy-measure-for-reducing-youth-unemployment-1640874982

[59] Akter H. Business Graduate-Ness and Work-Readiness: A Comparative Analysis of Public and Private Universities in Bangladesh. Asian Journal of University Education. 2020 Oct;16(3):59–77.

[60] Bin Rustam S. The Impact of Digital Marketing on Unemployment in Bangladesh. The Impact of Digital Marketing on Unemployment in Bangladesh, April 26, 2020.

[61] Dooley D, Catalano R, Hough R. Unemployment and alcohol disorder in 1910 and 1990: Drift versus social causation. Journal of occupational and organizational psychology. 1992 Dec;65(4):277 − 90.

[62] Grayson JP. Health, physical activity level, and employment status in Canada. International journal of health services. 1993 Oct;23(4):743 − 61. 10.2190/W5NR-A7A4-BX4A-T4F7 8276533

[63] Montgomery AL, Zarnowitz V, Tsay RS, Tiao GC. Forecasting the US unemployment rate. Journal of the American Statistical Association. 1998 Jun 1;93(442):478 − 93.

[64] Fliege H, Lee JR, Grimm A, Klapp BF. Risk factors and correlates of deliberate self-harm behavior: A systematic review. Journal of psychosomatic research. 2009 Jun 1;66(6):477 − 93. 10.1016/j.jpsychores.2008.10.013 19446707

[65] Chan J. A suicide survivor: the life of a C hinese worker. New Technology, Work and Employment. 2013 Jul;28(2):84–99.

[66] Haw C, Casey D, Holmes J, Hawton K. Suicidal intent and method of self-harm: A large‐scale study of self‐harm patients presenting to a general hospital. Suicide and Life‐Threatening Behavior. 2015 Dec;45(6):732 − 46. 10.1111/sltb.12168 25916308

[67] Anniko MK, Boersma K, Tillfors M. Sources of stress and worry in the development of stress-related mental health problems: A longitudinal investigation from early-to mid-adolescence. Anxiety, Stress, & Coping. 2019 Mar 4;32(2):155 − 67. 10.1080/10615806.2018.1549657 30465445

[68] Whisman MA, Baucom DH. Intimate relationships and psychopathology. Clinical child and family psychology review. 2012 Mar;15:4–13. 10.1007/s10567-011-0107-2 22124792

[69] Bagge CL, Glenn CR, Lee HJ. Quantifying the impact of recent negative life events on suicide attempts. Journal of abnormal psychology. 2013 May;122(2):359. 10.1037/a0030371 23088374

[70] Teo AR, Choi H, Valenstein M. Social relationships and depression: ten-year follow-up from a nationally representative study. PloS one. 2013 Apr 30;8(4):e62396. 10.1371/journal.pone.0062396 PMC3640036 23646128

[71] Wyder M, Ward P, De Leo D. Separation as a suicide risk factor. Journal of affective disorders. 2009 Aug 1;116(3):208 − 13. 10.1016/j.jad.2008.11.007 19128839

[72] Brassard, A., St-Laurent Dubé, M., Gehl, K., & Lecomte, T. (2018). Attachement amoureux, symptômes dépressifs et comportements suicidaires en contexte de rupture amoureuse [Romantic Attachment and Post Breakup Depression Symptoms and Suicidal Behaviour]. *Sante mentale au Quebec*, *43*(1), 145–162. 32338700

[73] The Daily Bangladesh. (2022, February 17). *Ex-RU student commits suicide after posting suicide note on FB*.The Daily Bangladesh.[ Internet] 2022, February 17.[ cited 2022, August,19].Available from: https://www.daily-bangladesh.com/english/education/69655

[74] BSEC official found dead in Dhaka with a “suicide note.” Bdnews24.com. Bd news 24.com[Internet]. 2022, May 2 [cited 2022, August, 19]. Available from:.https://bdnews24.com/bangladesh/bsec-official-found-dead-in-dhaka-with-a-suicide-note?fbclid=IwAR1p8RjcXa40aafXL_QiQjfKtf7eRzncQ5ZRxQytEVo0XC_PHLuL6YLft7c

[75] Brent DA, Johnson BA, Perper J, Connolly J, Bridge J, Bartle S, Rather C. Personality disorder, personality traits, impulsive violence, and completed suicide in adolescents. Journal of the American Academy of Child & Adolescent Psychiatry. 1994 Oct 1;33(8):1080-6. 10.1097/00004583-199410000-00003 7982858

[76] Mee S, Bunney BG, Reist C, Potkin SG, Bunney WE. Psychological pain: a review of evidence. Journal of Psychiatric Research. 2006 Dec 1;40(8):680 − 90. 10.1016/j.jpsychires.2006.03.003 16725157

[77] Brummett, B. H., Boyle, S. H., Siegler, I. C., Williams, R. B., Mark, D. B., & Barefoot, J. C. Ratings of positive and depressive emotion as predictors of mortality in coronary patients. *International journal of cardiology*, 2005; *100*(2), 213–216. 10.1016/j.ijcard.2004.06.016 10.1016/j.ijcard.2004.06.016 15823627

[78] Isometsä, E. T., Heikkinen, M. E., Marttunen, M. J., Henriksson, M. M., Aro, H. M., & Lönnqvist, J. K. The last appointment before suicide: is suicide intent communicated?. *The American journal of psychiatry*, 1995; *152*(6), 919–922. 10.1176/ajp.152.6.919 10.1176/ajp.152.6.919 7755124

[79] Mental Health: The Role of Media. The Daily Star[Internet.]2022, September 20. [cited 2022, August,19]. Available fromhttps://www.thedailystar.net/round-tables/news/mental-health-the-role-media-3123506

[80] Raleigh C. Political marginalization, climate change, and conflict in African Sahel states. International studies review. 2010 Mar 1;12(1):69–86.

[81] Klemenčič M, Park BY. 26. Student politics: Between representation and activism. Handbook on the politics of higher education. 2018:468 − 86.

[82] Altbach PG. Perspectives on student political activism. Comparative Education. 1989 Jan 1;25(1):97–110.

[83] Bangladesh democratic situation declining. The Daily prothom Alo [Internet]. 2021, April, 05[Cited 2023 Janurary 8]. Avaialable from:https://en.prothomalo.com/bangladesh/politics/bangladesh-democratic-situation-declining

[84] Mostofa SM, Subedi DB. Rise of competitive authoritarianism in Bangladesh. Politics and religion. 2021 Sep;14(3):431 − 59.

[85] Mostofa, S.M. Rise of Radical Nationalim in Bangladesh, South Asian Voices, 2023, https://southasianvoices.org/rise-of-radical-nationalism-in-bangladesh/

[86] Matthies-Boon V. Shattered worlds: political trauma amongst young activists in post-revolutionary Egypt. The Journal of North African Studies. 2017 Aug 8;22(4):620 − 44.

[87] da Costa S, Páez D, Martí-González M, Díaz V, Bouchat P. Social movements and collective behavior: an integration of meta-analysis and systematic review of social psychology studies. Frontiers in Psychology. 2023 Apr 21;14:1096877. 10.3389/fpsyg.2023.1096877 PMC10162496 37151317

[88] Charlson F, van Ommeren M, Flaxman A, Cornett J, Whiteford H, Saxena S. New WHO prevalence estimates of mental disorders in conflict settings: a systematic review and meta-analysis. The Lancet. 2019 Jul 20;394(10,194):240-8. 10.1016/S0140-6736(19)30934-1 PMC6657025 31200992

[89] Hanson RK. The psychological assessment of risk for crime and violence. Canadian Psychology/Psychologie canadienne. 2009 Aug;50(3):172.

[90] Hou J, Yu L, Fang X, Epstein NB. The intergenerational transmission of domestic violence: The role that gender plays in attribution and consequent intimate partner violence. Journal of Family Studies. 2016 May 3;22(2):121 − 39.

[91] Kim-Goh M, Suh C, Blake DD, Hiley‐Young B. Psychological impact of the Los Angeles riots on Korean‐American victims: Implications for treatment. American Journal of Orthopsychiatry. 1995 Jan;65(1):138 − 46. 10.1037/h0079590 7733209

[92] Ni MY, Yang L, Leung CM, Li N, Yao XI, Wang Y, Leung GM, Cowling BJ, Liao Q. Mental health, risk factors, and social media use during the COVID-19 epidemic and cordon sanitaire among the community and health professionals in Wuhan, China: cross-sectional survey. JMIR mental health. 2020 May 12;7(5):e19009. 10.2196/19009 PMC7219721 32365044

[93] Awami League leader committed suicide “unable to bear insult.” *The Daily Jugantor* [Internet] 2022, June 9, [cited 2022, August,19]. https://www.jugantor.com/country-news/560426/

[94] *BNP leader’s suicide!* DailyInqilabOnline[Internet]. 2019, October 11 [cited 2022, August,19]. Available from: https://m.dailyinqilab.com/article/240387/

[95] *Narcotics department identifies 3,500 drug dealers in Dhaka*. The Dhaka Tribune[ Internet]. (2021, September 24. [cited 2022, August, 19]. Available from https://archive.dhakatribune.com/bangladesh/dhaka/2021/09/24/narcotics-department-identified-3-500-drug-dealers-in-dhaka

[96] *Police: LSD seized during probe into DU student’s death*. The Dhaka Tribune [Internet]. 2021, May 2. [cited 2022, August, 19]. Available from https:https://archive.dhakatribune.com/bangladesh/dhaka/2021/05/27/police-lsd-seized-during-probe-over-du-student-s-death

[97] Borges G, Bagge CL, Cherpitel CJ, Conner KR, Orozco R, Rossow I. A meta-analysis of acute use of alcohol and the risk of suicide attempt. Psychological medicine. 2017 Apr;47(5):949 − 57. 10.1017/S0033291716002841 PMC5340592 27928972

[98] Randall JR, Nickel NC, Colman I. Contagion from peer suicidal behavior in a representative sample of American adolescents. Journal of Affective Disorders. 2015 Nov 1;186:219 − 25. 10.1016/j.jad.2015.07.001 26253902

[99] Youth commits suicide in Jashore - Countryside - observerbd.com.The Daily Observer[ Internet]. 2023, January 2. [cited 2022, August, 19]. Available from: https://www.observerbd.com/news.php?id=403520

[100] Brinkmann V, Zychlinsky A. Beneficial suicide: why neutrophils die to make NETs. Nature Reviews Microbiology. 2007 Aug;5(8):577 − 82. 10.1038/nrmicro1710 17632569

[101] *DU student dies after “jumping off” campus building*. The Financial Express [Internet] 2018, April 1, [cited 2022, August, 19]. Available from: https://thefinancialexpress.com.bd/national/country/du-student-dies-after-jumping-off-campus-building-1522563722

[102] Kazan D, Calear AL, Batterham PJ. The impact of intimate partner relationships on suicidal thoughts and behaviours: A systematic review. Journal of Affective Disorders. 2016 Jan 15;190:585 − 98. 10.1016/j.jad.2015.11.003 26583348

[103] Cheng Y, Zhang XM, Ye SY, Jin HM, Yang XH. Suicide in Chinese graduate students: a review from 2000 to 2019. Frontiers in psychiatry. 2020 Dec 4;11:579745. 10.3389/fpsyt.2020.579745 PMC7793914 33424658

[104] Garcia-Williams AG, Moffitt L, Kaslow NJ. Mental health and suicidal behavior among graduate students. Academic psychiatry. 2014 Oct;38:554 − 60. 10.1007/s40596-014-0041-y 24711096

[105] Miller NS, Mahler JC, Gold MS. Suicide risk associated with drug and alcohol dependence. Journal of addictive diseases. 1991 Jul 19;10(3):49–61. 10.1300/J069v10n03_06 1932152

